# How Many Wolves (*Canis lupus*) Fit into Germany? The Role of Assumptions in Predictive Rule-Based Habitat Models for Habitat Generalists

**DOI:** 10.1371/journal.pone.0101798

**Published:** 2014-07-16

**Authors:** Dominik Fechter, Ilse Storch

**Affiliations:** Freiburg University, Chair of Wildlife Ecology and Management, Baden-Württemberg, Germany; Institut Pluridisciplinaire Hubert Curien, France

## Abstract

Due to legislative protection, many species, including large carnivores, are currently recolonizing Europe. To address the impending human-wildlife conflicts in advance, predictive habitat models can be used to determine potentially suitable habitat and areas likely to be recolonized. As field data are often limited, quantitative rule based models or the extrapolation of results from other studies are often the techniques of choice. Using the wolf (*Canis lupus*) in Germany as a model for habitat generalists, we developed a habitat model based on the location and extent of twelve existing wolf home ranges in Eastern Germany, current knowledge on wolf biology, different habitat modeling techniques and various input data to analyze ten different input parameter sets and address the following questions: (1) How do a priori assumptions and different input data or habitat modeling techniques affect the abundance and distribution of potentially suitable wolf habitat and the number of wolf packs in Germany? (2) In a synthesis across input parameter sets, what areas are predicted to be most suitable? (3) Are existing wolf pack home ranges in Eastern Germany consistent with current knowledge on wolf biology and habitat relationships? Our results indicate that depending on which assumptions on habitat relationships are applied in the model and which modeling techniques are chosen, the amount of potentially suitable habitat estimated varies greatly. Depending on a priori assumptions, Germany could accommodate between 154 and 1769 wolf packs. The locations of the existing wolf pack home ranges in Eastern Germany indicate that wolves are able to adapt to areas densely populated by humans, but are limited to areas with low road densities. Our analysis suggests that predictive habitat maps in general, should be interpreted with caution and illustrates the risk for habitat modelers to concentrate on only one selection of habitat factors or modeling technique.

## Introduction

Large carnivores are currently recolonizing much of Europe [1], [2], [3]. Due to their large spatial requirements and that they compete for game and poach livestock, this recolonization comes with a high potential for human-wildlife conflicts [4]. To address these potential conflicts in advance, managers often use predictive habitat models to determine the amount and distribution of potentially suitable habitat and areas likely to be recolonized (for a review on different modeling techniques see [5], [6]). When field data for the study area in question are scarce, managers often turn to extrapolating results from other study areas or using rule-based models [7], also known as an expert based approach [8], [9], [10] or a knowledge based approach [11], [12], where verbal rules on wildlife-habitat relationships are replaced by equations, classifying areas into suitable or unsuitable habitat [7], [13]. Despite the fact that extrapolating results from different study areas or making general assumptions on wildlife-habitat relationships can lead to erroneous estimations on potentially suitable habitat [6], [14], [15], [16], these methods have been applied for a variety of species worldwide, including large carnivores (*Wolf* (*Canis lupus*): [17], [18], *Eurasian lynx* (*Lynx lynx*): [13], *Puma* (*Puma concolor*): [19], *Cheetah* (*Acinonyx jubatus*): [20], *Himalayan brown bear* (*Ursus arctos*): [21]. The more specialized a species is in its habitat requirements, the easier it is to differentiate between suitable and unsuitable habitat [22]. Habitat generalists are more difficult, because opportunistic habitat use may suggest different habitat preferences in different parts of their ranges. Wolves, for example, use a wide range of habitat types, but show certain preferences for forest cover in most parts of their range [23], [24], [25], [26]. In Europe, coexistence with humans in rural and even urban landscapes may explain why wolves may show pronouncedly different habitat associations in different countries. In Poland, for example, wolves use meadows and wetlands in addition to forest [26]. In Portugal, presence of wolves appears closely linked to livestock abundance rather than a certain land cover type [27], and in Russia wolves occupy mosaic habitats of forest and agricultural areas [28]. In Spain wolves frequently use agricultural fields [29], while in Italy and Romania wolves use shrub land and garbage dumps [23], [30], [31].

In the Lausitz region, NE Germany, a re-colonizing population is rapidly expanding [2], [32]. Because field data are limited, it is unavoidable to use assumptions about wolf-habitat relationships for predicting the further recolonization of Germany by wolves. These assumptions are based either on expert opinion or data from other populations (e.g., [27]) and have a strong influence on model outcome [33], [34].

In this paper, our primary objective is to investigate the uncertainty in rule-based habitat models for habitat generalists. Using the wolf as a model for habitat generalists, we developed a spatially-explicit, predictive rule-based habitat model and tested ten different model input parameter sets. In six model input parameter sets, suitable habitat was determined by land cover types and distance from roads and settlements, and in three model input parameter sets, suitable habitat was determined by density relationships between wolf habitat suitability, and road and human population densities. The tenth model input parameter set was a meta-model input parameter set, combining the results from the previous model input parameter sets to create a conservative overview map. For the ten model input parameter sets, we analyzed the availability of suitable habitat in the existing twelve Lausitz wolf pack home ranges and extrapolated these results, on a home range level, for all of Germany. We then used this analysis to address three questions related to habitat modeling and conservation planning for the wolf in Germany: (1) How do a priori assumptions and the selection of different input data or habitat modeling techniques affect the quantity, extent and arrangement of potentially suitable wolf habitat and the number of wolf packs in Germany? (2) In a synthesis across model input parameter sets, what areas can be expected to be the most suitable for wolves in Germany? (3) Are the location and extent of existing wolf pack home ranges in the Lausitz consistent with current knowledge on wolf biology and habitat relationships?

## Methods

### Study area and origin of data

As a study area for the habitat model, we used the entire area of Germany. The habitat model comprised a variety of geographically referenced environmental data. For general information on land use, we used CORINE (Coordination of Information on the Environment) Land Cover classification raster data (CLC2006), with a cell size of one hectare. For Germany, the data set, CLC2006, includes 37 different land cover types grouped in five main categories: (1) artificial surfaces, (2) agricultural areas, (3) forests and semi natural areas, (4) wetlands and (5) water bodies. Based on wolf habitat relationships (see below), we built three different ***land cover type data sets*** **: LCTS-A** (forest only), **LCTS-B** (forest and various types of open land) **and LCTS-C** (all areas which are not urban fabric) (Table 1). We obtained road data from the Open Street Map project, [35], and subsampled two ***road network data sets*** **: RNDS-T** (tertiary roads up to motorways) **and RNDS-NT** (secondary roads to motorways) (Table 1). Information on the human population at the community level was provided by the German Federal Agency for Cartography and Geodesy (BKG). We reclassified the number of inhabitants to human population density (inhabitants/km^2^) in a **human population density data set (HPDS)** (Table 1). To assess habitat suitability threshold values (see below) for our model, we used the location and extent of the twelve Lausitz pack home ranges in NE-Germany. The location and spatial extent of these home ranges, are estimations based on tracking data, camera traps and personal observations (I. Reinhardt, pers. comm.), and were supplied by LUPUS Wildlife Consulting.

**Table 1:** Environmental parameters used in the four rules of the habitat models for all model input parameter sets except the meta-model input parameter set COM, which was derived from the results of the other model input parameter sets and the connectivity analysis.

Environmental parameter	Definition/used data	
Land cover type data sets (LCTS)		
LCTS-A	Forests and transitional woodland/shrub (CLC-Code 311, 312, 313, 324)	
LCTS-B	Forests and transitional woodland/shrub, mineral extraction and dump sites, non-irrigated arable land, pastures, land principally occupied by agriculture with significant areas of natural vegetation, natural grassland, moors and heath land, sparsely vegetated areas, inland marshes and peat bogs (CLC-Code 131, 132, 211, 231, 243, 311, 312, 313, 321, 322, 324, 333, 411, 412)	
LCTS-C	All areas which are not urban fabric, industrial, commercial or transport units, as well as glaciers and marine wetlands and waters, respectively (i.e. everything but CLC-Code 111, 112, 121, 122, 123, 124, 335, 421, 422, 423 521, 522, 523)	
Road network data sets (RNDS)		
RNDS-NT	OSM-classification motorways, trunks, primary roads and secondary roads	
RNDS-T	All roads of RNDS-NT and in addition OSM-classification tertiary roads	
Human population density data set (HPDS)	Human population density at the community level (i.e. inhabitants/km^2^)	
Home range size	Home range size for all model input parameter sets was set to 200 km^2^	
Core areas	Unfragmented suitable habitat covering a minimum of 5% of the home range (i.e. 10 km^2^). In addition, home ranges of 10 and 15% (i.e. 20 and 30 km^2^) were analyzed	
Buffer zones	Areas surrounding roads and settlements (including urban areas) unsuitable for wolves. Buffer radii for roads: 0.25 km, 1 km and 2 km. Buffer radii for settlements: 0.5 km, 1 km and 3.5 km	
Railroad network data	OSM-classification rail and narrow gauge (only used in connectivity analysis)	
Rivers and streams	Navigable federal waterways (only used in connectivity analysis)	
Road density thresholds	**Road Density (km/km^2^)**	**Suitability class**
	0–0.23	3 (Highly suitable)
	0.23–0.6	2 (Suitably)
	0.6–1.2	1 (Marginally suitable)
	Over 1.2	0 (Not suitable)

Following Rykiel [36], we created two validation data sets based on documentation of wolf presence in Germany. An extensive internet search revealed over 5000 press releases from regional and national newspapers articles reporting wolf occurrences in Germany between 2009 and 2012, based on killed livestock or prey, snow and radio tracking data, observational data, data from camera traps and scat analysis. We filtered all reports for multiple entries using spatial location, timeframe and sometimes genetic information as criteria. Then, we categorized reported occurrences following the SCALP (Status and Conservation of the Alpine Lynx Population) criteria [37]. Only occurrences falling into categories C1 (hard facts) and C2 (confirmed) were geographically referenced using the reported location, or the geographic center of the area, and used for subsequent analysis. We created two validation data sets of wolf occurrence points. The first set was a subset of the second, and contained the locations of 17 resident solitary wolves or wolf packs outside the Lausitz area. The second validation data set also contained locations of non-resident wolves. Note that we included points in the Lausitz area in data set two, if the date of the occurrence was earlier than the establishment of a pack in the same region. We created a third data set with 250 random points throughout Germany and checked if mean habitat suitability at the validation points was significantly higher than at random points.

### Wolf habitat relationships

We modeled suitable habitat for wolves based on current knowledge of wolf habitat use. To account for habitat specialization in different geographical ranges, we built nine model input parameter sets (AT, ANT, BT, BNT, CT, CNT, T, NT and HP (Table 2)) using combinations of the different land cover type data sets (LCTS-A, LCTS-B or LCTS-C), road network data sets (RNDS-T or RNDS-NT) and information on human population density (HPDS) (Table 1). In the three land cover type data sets, the amount of potentially suitable land cover types increased from LCTS-A (forest only) over LCTS-B (forest and various types of open land) to LCTS-C (all areas which are not urban fabric). In the road network data sets, the number of road categories used in the analysis decreased from RNDS-T (tertiary roads up to motorways) to RNDS-NT (secondary roads to motorways). The model input parameter sets were built upon the following wolf habitat relationships:

**Table 2:** Overview of the ten model input parameter sets used for estimating habitat availability for wolves in Germany.

Model input parameter set ID	Model type	Data sets	Need for core areas included	Buffers included
AT	Rule based model	LCTS-A and RNDS-T	Yes	Yes
BT	Rule based model	LCTS-B and RNDS-T	Yes	Yes
CT	Rule based model	LCTS-C and RNDS-T	Yes	Yes
ANT	Rule based model	LCTS-A and RNDS-NT	Yes	Yes
BNT	Rule based model	LCTS-B and RNDS- NT	Yes	Yes
CNT	Rule based model	LCTS-C and RNDS- NT	Yes	Yes
T	Rule based model	RNDS-T	Yes	No
NT	Rule based model	RNDS-NT	Yes	No
HP	Rule based model	HPDS	No	No
COM	Synthesis model	Synthesis of all results from the above model input parameter sets	No	No

Notes: Model input parameter sets AT, BT, CT, ANT, BNT and CNT are combinations of a land cover type data set (LCTS) and a road network data set (RNDS). Model input parameter sets T, NT and HP contain only one data set, either a RNDS or the human population density data set (HPDS). The meta-model input parameter set, COM, is a synthesis of the results of all model input parameter sets. Due to the low spatial resolution, no core area could be determined for model input parameter sets HP and COM. Buffers for roads and settlements were only used in the first six model input parameter sets.

Although wolves are considered habitat generalists [38], [39], they are generally closely linked to forest cover [25]. We therefore assumed forest to be generally suitable for wolves in the model input parameter sets containing a land cover type data set (LCTS), i.e. AT, ANT, BT, BNT, CT and CNT (Table 1 and 2).

However, studies have shown that other land cover types including agricultural fields, garbage dumps or shrub lands can also provide suitable habitat [23], [28], [29], [30], [40]. We therefore assumed these habitat types to be suitable in the model input parameter sets using LCTS-B or LCTS-C, i.e. BT, BNT, CT and CNT (Table 1 and 2).

Different studies indicate that wolves avoid structures, such as roads and settlements [40], [41], [42], [43], [44], [45], [46], and an area of human activity that extends beyond the structure itself, thus reducing potentially suitable habitat surrounding the structure. This often called “buffer effect”, can range from 0.25 km up to 3.5 km from the border of the structure, depending on the level of disturbance [27], [45], [47], [48]. Although, there is evidence that avoidance could be temporal or spatiotemporal segregation [23], [47], [49], or behavior could change with habituation [44], [50], [51], [52], [53], so we included buffers for roads and settlements in the six model input parameter sets combining a land cover type data set and a road network data set, i.e. AT, ANT, BT, BNT, CT and CNT (Table 1 and 2).

Thiel [41] showed a negative correlation between road density and wolf habitat suitability and suggested a mean road density of 0.6 km/km^2^ as a threshold value for suitable habitat. Although threshold values may change over time (Table S1), we assumed this relationship to be valid. High road density facilitates accessibility of wolf territory to humans and thus the opportunity to kill wolves [54], [55]. We therefore calculated the road density (km/km^2^) in the two road network data sets RNDS-T and RNDS-NT and used the results for the model input parameter sets T and NT as the key explanatory variables (Table 1 and 2).

Wolves tend to establish their home ranges in areas with the least human disturbance [45], and human population density is considered to have a strong influence on wolf habitat suitability [24], [42], [43]. However, wolves may live in close proximity to areas with human activity [25], [52], [53], [56]. Fuller et al. [43] found that over 80% of wolf packs and solitary wolves in Minnesota (USA) inhabited areas with either <0.7 km roads/km^2^ and <4 humans/km^2^ or <0.5 km roads/km^2^ and <8 humans/km^2^, respectively. We addressed human population density as the key explanatory variable in the model input parameter set HP (Table 1 and 2).

Wolf home range size is dependent on factors such as prey species and abundance [57], wolf pack size [56], [58], [59], population density [56], [58], [60] and population status [61]. Home range sizes for wolves in Europe within the latitudinal range of 42°–53° North, where the main prey species are red deer (*Cervus elaphus*), roe deer (*Capreolus capreolus*) and wild boar (*Sus scrofa*), vary between 87 km^2^ and 243 km^2^, with a mean of 170 km^2^ (see [62] for a review). The mean home range size, calculated from the twelve estimated wolf pack home ranges in the Lausitz, is approximately 215 km^2^. For our model we assumed a home range size of 200 km^2^ (Table 1).

In Europe, core areas, i.e. areas without human settlements and with low road densities within wolf home ranges, vary between 3.3 km^2^ and 28 km^2^, representing 2.3% to 15% of the total home range size [23], [61], [62], [63]. In our model, we assumed core areas needed to be a minimum of 5% for each 200 km^2^ home range, i.e. 10 km^2^ (Table 1).

### Modeling strategy

We built a spatially-explicit, predictive rule-based model [7], [13] and analyzed a total of ten different model input parameter sets to determine the quantity, extent and arrangement of areas potentially suitable as wolf habitat at the spatial level of a home range. We analyzed six model input parameter sets with combinations of the three different land cover type data sets (LCTS-A, LCTS-B and LCTS-C) and the two road network data sets (RNDS-T and RNDS-NT) (Table 1), resulting in model input parameter sets AT, BT, CT, ANT, BNT and CNT (Table 2) and three model input parameter sets using the relationship between wolf habitat suitability and road and human population density, as the key explanatory variable [41], [42]. Out of the three model input parameter sets where density was the key explanatory variable, two used road density (km/km^2^) calculated from the two RNDS, resulting in the model input parameter sets T and NT. One model input parameter set used human population density (inhabitants/km^2^) from the human population density data set, resulting in the model input parameter set HP (Table 2). In a meta-model input parameter set, we combined the results of all model input parameter sets to create an overview model input parameter set (model input parameter set COM) (Table 2).

Finally, we compared the two validation point data sets with the random point data set to test if the habitat models correctly predicted wolf occurrence in Germany; i.e., if mean wolf habitat suitability at wolf occurrence points was significantly higher than wolf habitat suitability at random points.

For all GIS operations we used ArcGIS (Version 10.0 by ESRI, Redlands, California, USA), while for statistical analysis we used R (Version 2.15.2).

### Identifying areas suitable as pack home ranges and determining pack numbers

#### The rule based model

We used the following rules to determine suitable wolf habitat in the six model input parameter sets using LCTS and RNDS (Table 2).

#### Rule 1

**Figure 1:**
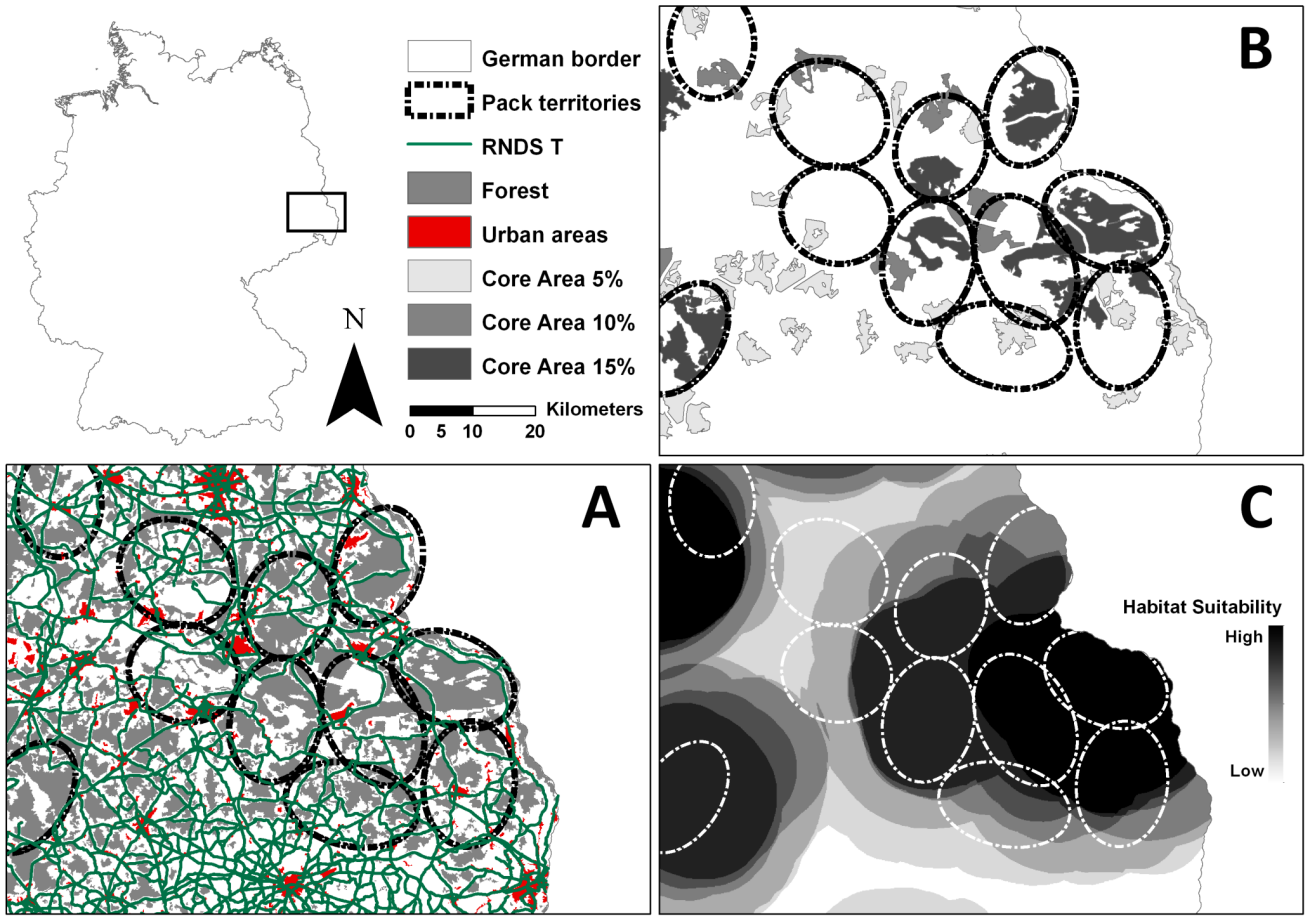
Application of the model rules. Maps A–C depict part of the Lausitz wolf area in NE Germany, illustrating the application of the rules used in the rule based model for modeling wolf habitat availability in Germany. Map D shows the baseline map for the connectivity analysis. Pack territory locations (dashed lines in black and white) show a first visual assessment of plausibility. (A) The first step was to apply model input parameter set rules 1 & 2 to a land cover map and a buffer set; here, model input parameter set AT (land cover types forest and transitional woodland/shrub, as well as roads, including tertiary roads) with buffer sets of 250 meters for roads and 500 meters for urban areas, used as an example. Suitable areas (in AT: forest and transitional woodland/shrub) are shown in grey; green lines indicate roads, urban areas are in red. The buffers have already been subtracted and are not shown. (B) Core areas in model input parameter set AT with the same buffer set for roads and urban areas as in A. The darker the area, the bigger the core area patch. (C) Resulting map of potentially suitable wolf habitat in model input parameter set AT. The darker the area, the more suitable the potential wolf habitat.

*Suitable land cover* - Only land cover types defined by the model input parameter sets LCTS were considered suitable (Table 1; Fig. 1A).

#### Rule 2

*Buffers* – Roads and settlements (including urban areas) reduce the amount of suitable habitat. We used three different buffers for roads and settlements, respectively. By pairing each road buffer with each settlement buffer, we created nine different buffer sets, which were subtracted from the LCTS, creating nine different model input parameter subsets (Fig. 1A). As a result, areas within the smallest buffer radii were not considered suitable in any of the nine buffer sets. However, areas within the buffer ranges of 1 km–2 km for roads or 1 km–3.5 km for settlements, were considered suitable in six out of nine buffer sets, leading to a declining disturbance effect.

#### Rule 3

*Minimum and mean suitable habitat requirements and home ranges –* A home range must have a minimum amount of suitable habitat. To determine the minimum and mean percentage of area covered by suitable habitat types within the twelve pack home ranges, we performed a zonal statistical analysis (internal function of ArcGIS 10.1) using the twelve Lausitz pack home ranges and the nine model input parameter subsets obtained by rule 2. For each 100*100 meter cell in Germany, we then calculated the amount of potentially suitable habitat in a radius of 8 km, representing an average home range size of 200 km^2^. A cell was considered marginally suitable if it reached the minimum amount of suitable habitat, and suitable if it reached or exceeded the mean amount of suitable habitat. Cells below the minimum value were considered unsuitable.

#### Rule 4

*Fragmentation and* c*ore areas* – Each wolf home range (200 km^2^, see rule 3) must have an unfragmented core area of at least 5%, i.e. 10 km^2^. To exclude highly fragmented areas we looked for unfragmented patches with a minimum of 10 km^2^ within the nine model input parameter set subsets created by the second rule acting as core areas. In addition, we also checked for core areas with 10% and 15% of the home range size (20 km^2^ and 30 km^2^) to give a higher weight to areas with larger unfragmented patches. After the application of rule 3, we checked each 100*100 meter cell in the model input parameter subsets, to determine if a core area was within the minimum home range area of 8 km radius. Cells which did not fulfill the requirements were excluded from the map created by rule 3 (Fig. 1B).

All remaining cells in the nine model input parameter subsets qualified to be the center of a potential home range with 200 km^2^. To obtain the real extent of potentially suitable habitat on a home range scale, we buffered the remaining cells in each model input parameter subset with an 8 km radius. The model input parameter subsets were joined together to reconstruct the original model input parameter set. The result was then reclassified, creating a map of potentially suitable wolf habitat on a home range level in seven suitability classes, ranging from 0 to 6, with 6 being the best possible habitat (Fig. 1C).

The three model input parameter sets using road density and human population density as key explanatory variables (i.e. model input parameter sets T, NT and HP, respectively) only used rule 3 and 4, since no land cover type data sets (LCTS) and buffers were needed. We used the following steps to determine suitable wolf habitat in the three model input parameter sets: for the model input parameter sets T and NT, we reclassified the road density (km/km^2^) for RNDS-T and RNDS-NT into four different suitability classes (Table 1). We then followed rule 3 and rule 4 to check for minimum and mean suitable habitat requirements, home range sizes, low fragmentation and core areas. The zonal statistic for rule 3 was performed with the twelve Lausitz pack home ranges and the suitability classes derived from the road density analysis (see above). Only unfragmented cells with suitability class 3 were used to determine a potential core area, while the remaining cells were buffered with 8 km to show the full extent of suitable wolf habitat and reclassified into the seven suitability classes according to the rule based model.

The model input parameter set, HP, was developed using only rule 3 of the rule based model. Due to limited spatial resolution at the community level, all patches of potentially suitable habitat exceeded the extent of a home range (200 km^2^). Therefore fragmentation was not an issue and we could not clearly differentiate core areas. Thus we discarded rule 4, buffered the cells according to the other models with 8 km and reclassified the model input parameter set to fit the seven suitability classes.

To create the meta-model input parameter set, COM, and identify areas determined as potentially suitable in all model input parameter sets, we pooled all data derived from the nine model input parameter sets for each cell. A cell was considered not suitable (suitability class 0) if it was unsuitable in at least a single model input parameter set. For all other cells, the value was determined by the mode value, i.e. the new value of the cell was based on the values occurring most often within all nine model input parameter sets, thus creating a conservative overview map for potentially suitable wolf habitat in Germany (COM).

For each model input parameter set, we calculated the number of wolf packs that could live in a given habitat patch by dividing the available potentially suitable habitat by an average home range size of 200 km^2^.

### Model validation

All points in the three validation data sets were buffered with a radius of 8 km, representing an average home range size of 200 km^2^. The location and extent of the home ranges are subject to change over time, therefore the points we assumed as the center of the home ranges may not be correct at different points in time. By buffering the points, we reduced potential location errors. In addition, our model predicts habitat suitability on a home range level. By using the extent of a home range, we ensured that our validation was performed at the home range level. For each of the 10 habitat model input parameter sets, we calculated the mean habitat suitability at all buffered points for the three validation data sets and analyzed them using Kruskal-Wallis and Mann-Whitney tests.

### Density and parameter correlation in the Lausitz pack home ranges

We calculated mean road and mean human population density in the twelve Lausitz pack home ranges for the data sets RNDS-T, RNDS-NT and HPDS, to determine whether they exceed the thresholds proposed by current knowledge on wolf habitat relationships (Table S1). We also looked for correlations between mean road and mean human population densities, and percent forest cover in the twelve Lausitz wolf pack home ranges.

## Results

### Model input parameter sets and potentially suitable habitat

**Figure 2:**
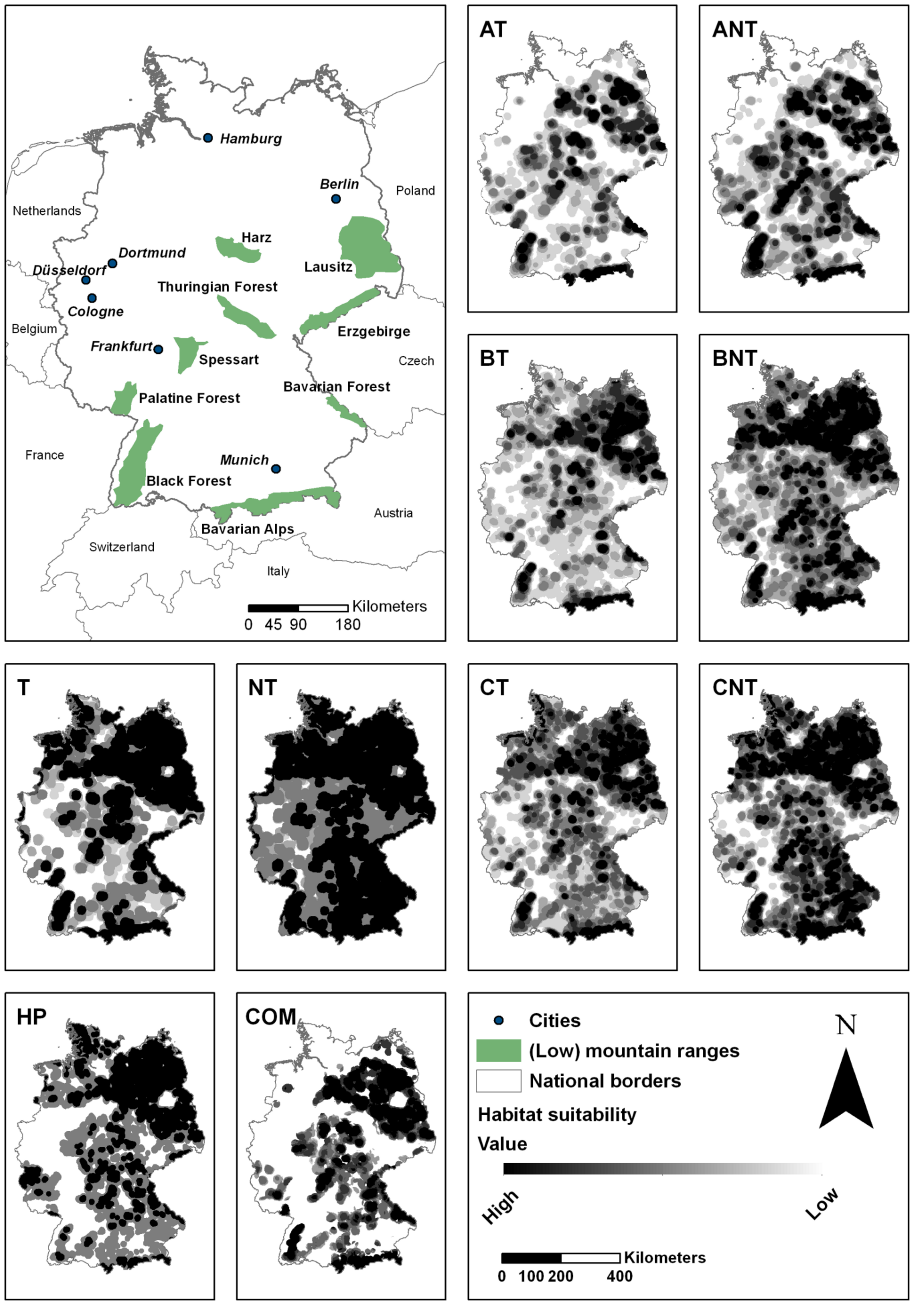
Wolf Habitat suitability maps for the ten model input parameter sets (small boxes). Top left corner: Orientation map to (low) mountain ranges (in green) and larger cities (black dots) in Germany and surrounding countries. The darker the area in the model input parameter set maps, the higher the habitat suitability. All model input parameter sets, except model input parameter set HP, consist of 7 suitability classes. Model input parameter set HP consist of only 3 suitability classes, because no core area could be identified. Habitat suitability maps were generated by successive application of the predefined rules.

For each of the ten model input parameter sets, the successive application of the four rules identified areas of potential wolf habitat in Germany (Fig. 2). Habitat suitability scores ranged from 0 (not suitable) to 6 (highly suitable) for the model input parameter sets: AT, BT, CT, ANT, BNT, CNT, T, NT, COM, and reached 0, 3 or 6 for the model input parameter set HP. All model input parameter sets were in accordance, that the most potentially suitable habitat is located in the east and north-east of Germany, areas with similar characteristics as the Lausitz (i.e. low road and human population density and similar land cover types). Other areas with high amounts of potentially suitable habitat were low mountain ranges such as the Bavarian Forest, Black Forest, Harz, Thuringian Forest, Spessart, and Bavarian Alps. The densely populated areas around the Ruhr district (triangle between Dortmund, Düsseldorf and Cologne), Berlin, Hamburg, Munich and Frankfurt were not considered suitable habitat in any of the ten model input parameter sets. Replacing the RNDS-T with the RNDS-NT parameter for the model input parameter sets AT, ANT, BT, BNT, CT, and CNT increased the amount of highly suitable habitat between 46%–125% (Table 4). The pooled data in the meta-model input parameter set COM revealed a concentration of potentially suitable habitat in the east and north-east of Germany, as well as in the low mountain ranges and Bavarian Alps. The west and north-west of Germany was not rated as suitable but consisted of small isolated patches of potentially suitable habitat (Fig. 2).

**Table 4:** Mean wolf habitat suitability in the ten model input parameter sets at the two validation data sets and random points.

	Validation data set one		Validation data set two		Random points data set					
	(Validation points, only resident wolves N = 17)		(Validation points including Non-resident wolves N = 155)		(Random points N = 250)		Kruskal-Wallis test	Mann-Whitney test		
Model input parameter set	Mean habitat suitability	SD	Mean habitat suitability	SD	Mean habitat suitability	SD		Data set 1 and 2	Data set 1 and 3	Data set 2 and 3
AT	4.0	2.3	3.5	2.0	1.8	1.9	p<0.001	p = 0.168	p<0.001	p<0.001
BT	4.6	1.9	4.0	2.0	2.5	1.9	p<0.001	p = 0.115	p<0.001	p<0.001
CT	4.9	1.7	4.3	1.9	3.1	1.9	p<0.001	p = 0.122	p<0.001	p<0.001
ANT	4.5	2.0	4.2	1.9	2.4	2.1	p<0.001	p = 0.275	p<0.001	p<0.001
BNT	5.2	1.5	4.6	1.6	3.7	1.8	p<0.001	p = 0.046	p<0.001	p<0.001
CNT	5.2	1.7	4.5	1.8	3.8	2.0	p<0.001	p = 0.072	p<0.001	p<0.001
T	5.1	1.5	5.1	1.7	3.7	2.2	p<0.001	p = 0.862	p = 0.003	p<0.001
NT	5.7	0.9	5.5	1.1	4.9	1.5	p<0.001	p = 0.353	p = 0.012	p<0.001
HP	5.0	1.8	4.8	1.8	3.3	2.2	p<0.001	p = 0.540	p<0.001	p<0.001
COM	4.4	2.5	4.5	2.1	2.3	2.3	p<0.001	p = 0.475	p<0.001	p<0.001

Note: Maximum value for mean wolf habitat suitability is 6.0.

### Pack numbers and pack size

For each model input parameter set, we calculated the potential number of wolf pack home ranges for Germany (Table 3). Depending on the model input parameter set, Germany could accommodate between 154 and 1769 wolf packs and 616–8845 wolves, assuming an average pack home range size of 200 km^2^ and an average pack size of 4–5 wolves [61] [57].

**Table 3:** Amount of potentially suitable wolf habitat for Germany by suitability class (in km^2^), and the range of potential wolf packs in the ten model input parameter sets.

	Habitat suitability class							
Model input parameter set	0	1	2	3	4	5	6	Number of packs (min – max)
AT	133609	69526	45869	32615	20397	30642	30729	154–1149
BT	57657	87705	62283	46853	28324	31261	49304	247–1529
CT	41865	46684	41020	64420	65233	34400	69764	349–1607
ANT	99054	53197	42703	49584	28771	30422	59656	298–1322
BNT	24148	27045	51194	77778	38425	33671	111126	556–1696
CNT	41609	26801	20289	52502	50474	43377	128335	642–1609
NT	9648	785	6502	90443	778	644	254590	1273–1769
T	58285	21479	40342	61013	6614	1895	173791	869–1525
HP	98856	0	0	121693	0	0	142838	714–1322
COM	130490	59337	580	13765	37992	42865	78359	392–1165

Note: Range of potential wolf packs from the number of potential wolf packs in the highest suitability class (6) to the lowest suitability class (1). Suitability class 0 provides no potentially suitable habitat.

### Model validation

To quantify the accuracy of the ten model input parameter sets, we calculated mean habitat suitability at all locations from the two validation data sets and the random points (Table 4). In both validation data sets, as well as at the random points, the model input parameter set NT had the best mean habitat suitability (5.7±0.9 for validation data set one, 5.5±1.1 for validation data set two and 4.9±1.5 for the random points). The overall mean habitat suitability for the validation data set one had a minimum mean habitat suitability of 4.0±2.3. As expected, the mean habitat suitability values for validation data set two were lower because these points included locations of non-resident individuals. Minimum mean habitat suitability was 3.5±2.0 in the model input parameter set AT. Mean habitat suitability at validation data set points was highly correlated with the availability of potentially suitable habitat in the highest suitability class (Pearson correlation r = 0.849, p<0.002, df = 8). The correlation between mean habitat suitability at random points and the availability of potentially suitable habitat was higher than for the validation data set points (Pearson correlation r = 0.914, p<0.002, df = 8). We performed a Kruskal-Wallis test for all model input parameter sets and paired the three data sets for each model input parameter set for a Mann-Whitney test (Table 4). In all model input parameter sets, mean habitat suitability for both validation data sets was significantly higher than for the random points (p<0.05). Within both validation data sets, mean habitat suitability differed, but not significantly (Table 4).

### Road density and human population density in the Lausitz pack home ranges

**Figure 3:**
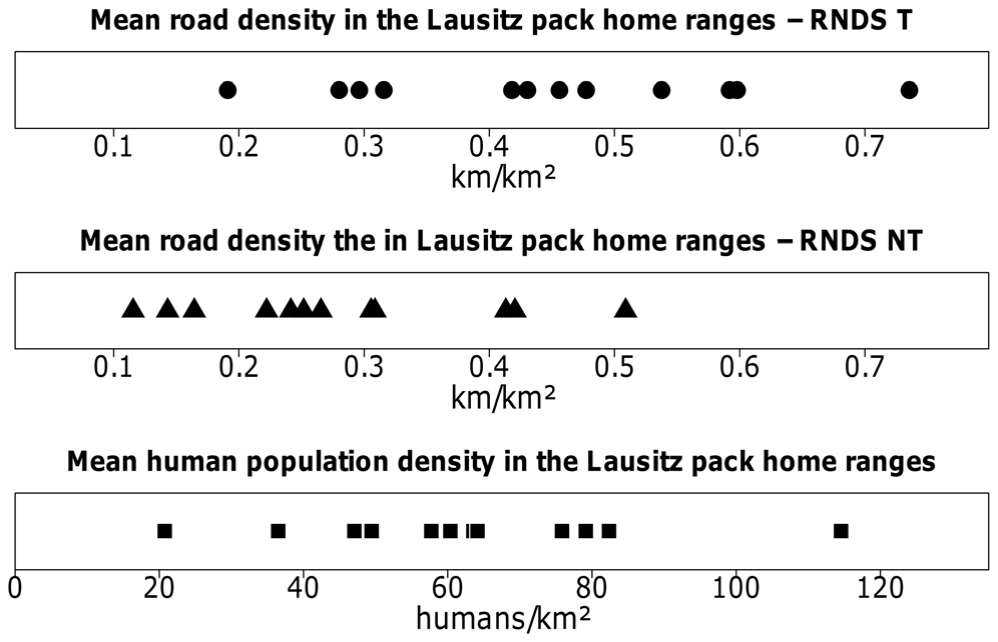
Mean road density and mean human population density in the twelve Lausitz wolf pack home ranges in model input parameter sets T, NT and HP. Each dot represents one wolf pack home range in the Lausitz. Mean road densities in the Lausitz in NE-Germany, for a home range area of 200 km^2^, range up to 4.6 km/km^2^ in model input parameter set T and 3.6 km/km^2^ in model input parameter set NT. Mean human population density for a home range area of 200 km^2^ could reach 2622 humans/km^2^.

For the model input parameter sets T, NT and HP, we calculated road and human population density in the twelve Lausitz pack home ranges (Fig. 3). Mean road density in the pack home ranges ranged from 0.12 km/km^2^ in the model input parameter set NT up to 0.74 km/km^2^ in the model input parameter set T. Only one pack home range had a mean road density which exceeded the proposed threshold of 0.6 km/km^2^ suggested by Thiel [41] (Table S1). Pack home ranges included cells with a road density up to 4 km/km^2^ in model input parameter set T and 2.49 km/km^2^ in model input parameter set NT. Mean human population density in Lausitz wolf pack home ranges in model input parameter set HP, ranged from 20 humans/km^2^ to 115 humans/km^2^ (Fig. 3) and exceeded the threshold from Fuller et al. [43] by a multiple (Table S1). The highest calculated human population density in one cell of a pack home range was 380 humans/km^2^.

### Correlations between parameters in the Lausitz pack home ranges

We checked for correlations between road density (in RNDS-T and –NT), human population density and percent forest cover in the twelve Lausitz pack home ranges (Table 5). Road density and human population density were strongly correlated. Percent forest cover was less strongly correlated to human population density and RNDS-NT than to RNDS-T.

**Table 5:** Pearson's correlations between parameters of road density, human population density and percent forest cover for the Lausitz wolf pack home ranges (N = 12).

Parameter	r =	p =
RNDS-T vs. RNDS-NT	0.79861	0.0018
RNDS-T vs. human population density	0.74331	0.0056
RNDS-NT vs. human population density	0.78204	0.0027
RNDS-T vs. %forest cover	−0.7754	0.0031
RNDS-NT vs. %forest cover	−0.5698	0.0531
Human population density and %forest cover	−0.6082	0.0359

## Discussion

Our results show that the a priori assumptions made on habitat relationships and the selection of different input data or different modeling techniques have a tremendous influence on the model outcome [33], [34]. In the case of a habitat generalist like the wolf, where opportunistic habitat use makes it difficult to distinguish between areas suitable or unsuitable as habitat, the results vary even more. Depending on the combination of *land cover type data set* (LCTS), road *network data set* (RNDS) or *human population density data set* (HPDS) the quantity, extent and arrangement of potentially suitable habitat can vary by more than 800% from one model input parameter set to another. Since all model input parameter sets were based on field studies, habitat modelers risk questionable assumptions and erroneous suitable habitat estimations by concentrating on only one selection of habitat factors or one modeling strategy

However, analyzing different model input parameter sets within the full range of variation of species -habitat relationships can provide helpful initial information about the range in area of potentially suitable habitat and resultant wolf and wolf pack numbers. Model input parameter set AT, with the least amount of potentially suitable habitat, provided enough suitable habitat for a minimum of 154 wolf packs in all of Germany, i.e. roughly 600 wolves [61]. Due to the LCTS-A (only forests and transitional woodland/shrub), RNDS-T and need for a core area, this model input parameter set restricts wolves to large unfragmented forest areas. Because wolves are known to live in non-forested areas [24], [29] this number should be interpreted as a lower limit. Model input parameter set NT predicted the highest amount of suitable habitat resulting in a potential for more than 1250 wolf packs and 5000 wolves in the best suitability class 6. Accounting for the numerous anthropogenic deaths by illegal shooting [1], this number seems unrealistic and it is more likely that human attitude will restrict wolf presence, rather than the availability of suitable habitat [39], [64].

We used the results from the first nine model input parameter sets to create the meta-model input parameter set, COM. This allowed us to filter the model input parameter sets and extract the commonalities of all model input parameter sets. Model input parameter set COM was mostly influenced by model input parameter sets AT and HP, thus augmenting the lower limit created by model input parameter set AT by an anthropogenic factor and increasing the suitability in areas sparsely populated by humans. The north-east of Germany and the lower mountain ranges had the largest unfragmented patches of potentially suitable habitat, areas with similar landscape and land use characteristics as the Lausitz.

Existing wolf habitat models (Knauer et al, unpublished data, [27]) predict suitable habitat for 400–441 wolf packs in Germany. Although the total number of predicted wolf packs differs only by 10%, the location and extent of the suitable patches is different in both habitat models. Depending on the a priori model assumptions, some large forest areas like the Bavarian Forest are classified as very suitable habitat in one model (Knauer et al., unpubl.) and as poor habitat in the other [27]. The sparsely populated north-eastern part of Germany, dominated by agricultural land and small forest patches leads to a higher quality of potential wolf habitat in the model by [27]. In addition, definitions on whether small or isolated patches are included increases the number of potential wolf packs by up to 100%. This demonstrates the strong influence of modeling strategies on the model result and the importance of their careful interpretation.

The validation data sets showed a high consistency for all model input parameter sets in our model. Although not significant, throughout all model input parameter sets, mean habitat suitability at the validation points of data set one (only residential wolves) was higher than those from data set two (including non-residential wolves). This is not surprising, given that the points of validation for data set two included non-resident and dispersing wolves. Dispersing wolves have been known to pass through large areas of unsuitable habitat, but they use suitable habitat whenever available [39], thus leading to an interdependency of the two data sets. Mean habitat suitability values at the validation points for both data sets were significantly higher than mean habitat suitability values at the random points. The high consistency for model input parameter set NT could be either based on a good model fit, or the correlation between availability of best suitable habitat and mean habitat suitability at the validation points, masking the congruity. However, the correlation between availability of best suitable habitat and mean habitat suitability at the random points was even higher, but validation data set one and the random points differed significantly. This indicates that the high consistency for our model input parameter sets cannot be reduced to the correlation mentioned above.

According to current knowledge, road density and human population density are considered good indicators of wolf habitat suitability [23], [41], [42], [61]. However, high road density could become less of a factor in wolf habitat selection as human attitude towards wolves improves [41], [65]. We analyzed the Lausitz pack home ranges regarding these factors to see if they are consistent with the current state of research. For all but one of the pack home ranges, road density of the RNDS-T and RNDS-NT was below the threshold of 0.6 km/km^2^ suggested by Thiel [41]. The pack home range exceeding the threshold had a mean road density of 0.74 km/km^2^ which was still considered suitable in other studies [46], [66], [67]. This gives a strong indication, that the threshold by Thiel [41] is transferable to the German wolf population. The mean human population density in the Lausitz pack home ranges exceeded the threshold from Fuller et al. [43] by a multiple. This may provide evidence that tolerance towards humans in densely populated areas might be higher than expected. However, compared to all possible locations of home ranges in the Lausitz, the existing pack home ranges favored areas with low road and human population density over other densely populated areas.

Most field studies conclude that road density, human population density or forest cover are the key variables associated with wolf occurrence and wolf habitat suitability [23], [26], [40], [41], [42], [43], [44], [45], [46], [68]. For the Lausitz pack home ranges, these variables were highly correlated. Therefore, the habitat suitability maps generated by our models have the tendency to classify some areas in Germany as suitable habitat throughout all model input parameter sets. Although each of the variables might be applicable to predict wolf habitat suitability, reducing the probability of wolf occurrence by using only one of these variables neglects the fact that all these variables are surrogates for an interdependent cluster of factors, all of which needs further investigation.

Transferring field data or extrapolating results from other study sites or other geographic regions is always difficult and has a great potential for error [6], [16], [69]. Nonetheless, when field data for a study area are limited, then using data from other areas can serve as a substitute. When substituting data, it is important to test various model input parameter sets, incorporating the full range of data. This is especially important in the case of a habitat generalist such as the wolf [38], [39], whose opportunistic habitat use make it difficult to exclude a certain habitat type from the list of potentially suitable habitat. Doing so, one can determine the whole spectrum of potentially suitable habitat. Therefore, we included data on wolf-habitat relationships from various study areas in Europe and North America.

In our model we analyzed the composition and amount of potentially suitable habitat according to our model input parameter sets within the twelve Lausitz pack home ranges, thereby creating thresholds which determine the requirements for potentially suitable habitat at the home range level in Germany. With these thresholds, we were able to extrapolate the results from the Lausitz to the whole of Germany, allowing us to use the full range of data on wolf habitat relationships, while still minimizing the geographical extrapolation error [16].

### Possible shortcoming of our modeling approach

Uncertainty in data and model assumptions can have a strong influence on the results and possibly lead to misinterpretation [13]. Therefore, it is important to document every step and list possible shortcomings of the model in order to reduce the potential for misinterpretation. Although the location and extent of the Lausitz pack home ranges are based on radio tracking data, camera traps and direct observations (I. Reinhardt, pers. comm.), they are still estimations and therefore prone to errors. However, since all field studies face the problem of using limited spatial data to estimate home ranges, we presume this to be acceptable. For our analysis, we assumed a home range size of 200 km^2^, based on the mean home range size of the Lausitz pack home ranges (ca. 216 km^2^) and the results from Findo et al. [62] (average home range size from 10 different wolf packs in Europe ca. 170 km^2^). Therefore we might have over or underestimated the number of wolves and wolf packs by up to 20%.

Mech [39] and Peterson [64] suggested that wolves require adequate prey abundance and reduced killing by humans more than wilderness to survive. Wolf diet strongly depends on the availability of prey species [70]. In Europe [71], [72], [73], as well as in North America [74], [75], [76], the preferred prey species are large wild ungulates. For the Lausitz population in NE-Germany, the dominating prey species is roe deer (*Capreolus capreolus*) (55.3%), followed by red deer (*Cervus elaphus*) (20.8%) and wild boar (*Sus scrofa*) (17.7%) [70]. If the abundance of wild ungulates is limited, wolves are able to adapt and shift their diet towards more accessible food sources, often livestock or waste [23], [77], [78]. We decided not to include prey density, i.e. wild ungulate density, in our models for several reasons. First, information on absolute prey density is not available and we would have to rely on estimations from the yearly hunting bag. Although this is a standard method, there are numerous factors influencing hunting success and thus the size of the hunting bag [79]. Secondly, recent studies have shown no negative influence on the hunting bag for wild ungulates in hunting districts in the Lausitz, where wolves have lived for the past 8 years [70]. Ungulate densities, especially for roe deer and wild boar, have increased during the last 50 years in Germany [79], [80]. Therefore we see no strong evidence for prey density being a limiting factor at the moment. This may be an important consideration in the future, if ungulate densities were to decline, but wolves are known to shift their feeding habits opportunistically [77], [81], [82] and a decline in ungulate densities may have little impact.

The OSM project offers no guarantee that the data set is accurate and complete. The OSM data set [35] is still changing every day, increasing in detail and accuracy. Ludwig et al. [83] showed that accuracy on a national level in Germany was already high in a data set one year older than the one we used; therefore we are confident that using an updated OSM data set would only lead to minor changes. However, accuracy and level of completeness of data might vary between countries, rendering OSM unsuitable for habitat modeling within and between countries.

In the model input parameter sets T and NT, we used only the RNDS as explanatory variables, leading to an overestimation of potentially suitable habitat. Urban areas were not necessarily categorized as unsuitable, because roads smaller than a tertiary road were not included in the RNDS. As a result, large cities like Munich or Hamburg contained large amounts of fairly suitable habitat (Fig. 2, model input parameter set T and NT).

### Conclusions and guidelines for further research

It was not our goal to create an indisputable habitat model for wolves in Germany, but to show how different assumptions lead to dramatically different results. Conservation managers often try to derive initial information on future species distribution from rule based models in order to prepare and make decisions before the target species colonizes an area and human-wildlife conflicts arise [13], [84]. If information on habitat requirements derived from local field studies are scarce or not available, rule based habitat models can provide an opportunity to estimate potentially suitable habitat [13]. The more specialized a species is in their habitat requirements, the more accurate a rule based habitat model can be. In the case of a habitat generalist like the wolf, there are a number of plausible model input parameter sets, leading to very different estimations of potentially suitable habitat. Nonetheless, a detailed analysis of these different model input parameter sets in the full range of variation of species-habitat relationships can give an initial understanding on the quantity and arrangement of potentially suitable habitat. As of 2011, the German wolf population consisted of 17 wolf packs or scent marking pairs and several solitary wolves, leading up to a minimum of 43 adult wolves [2]. Our analysis suggest that Germany comprises enough highly suitable habitat for a minimum of 154 wolf packs, but that around 400 wolf packs seem reasonable, provided that key habitat in Central Germany remains accessible and the public attitude regarding wolves is positive [65], [85].

We suggest conducting a thorough population viability analysis [86] using a spatially explicit individual based population model to further investigate the expansion of the population to the west and south of Germany. By doing so, the population must be monitored intensively to collect sufficient data on survival and reproductive parameters, dispersal behavior and spatial requirements. We emphasize that a rule based habitat model must be interpreted with caution, as it is based on assumptions. Although these assumptions are based on the current best understanding of the species' biology, they are extrapolations, and therefore, always liable to misinterpretation [87]. Our modeling approach is not restricted to the wolf and could be used for other habitat generalists as well.

## Supporting Information

Table S1 Mean road density in a home range for long term wolf survival in different study areas, based on [88], expanded with additional field studies.
(DOCX)
