# Supplementary material for: How Many Wolves (Canis lupus) Fit into Germany? The Role of Assumptions in Predictive Rule-Based Habitat Models for Habitat Generalists
Source: PLoS One. 2014 Jul 16;9(7):e101798. doi: 10.1371/journal.pone.0101798 (PMC4100756; doi:10.1371/journal.pone.0101798)
Supplement: Table S1 — Mean road density in a home range for long term wolf survival in different study areas, based on [88], expanded with additional field studies. (DOCX) [file pone.0101798.s001.docx]

Table S1. Mean road density in a home range for long term wolf survival in different study areas, based on [88], expanded with additional field studies.

| Road Density Threshold (km/km²) | Study Area | Citation |
| --- | --- | --- |
| 0.23 (coreareas) / 0.45 | USA (Wisconsin/Michigan) | [17] |
| 0.5 (<8 humans/km²) | USA (Minnesota) | [43] |
| 0.58 | USA (Minnesota) | [42] |
| 0.6 | USA (Wisconsin ) | [41] |
| 0.63 | USA (Wisconsin) | [89] |
| 0.7 (< 4 humans/km²) | USA (Minnesota) | [43] |
| 0.73 | Minnesota | [66] |
| 1.0 | Canada (JasperNational Park) | [46] |
| 1.2 | Poland (Białowieża) | [67] |
| 1.4 | USA (Minnesota) | [90] |

References

1. Switalski TA (2006) How Many is Too Many: A Review of Road Density Thresholds for Wildlife. Winter Solstice 11(4): 6 – 8.
2. Kohn BE, Thiel RP, Hansen JL (2001) Road density as a factor in habitat selection by wolves and other carnivores in the Great Lakes Region. Carnivore Conservation in the Twenty-first Century 97, 18(4): 110.
3. Merrill SB (2000) Road densities and gray wolf, Canis lupus, habitat suitability: an exception. Can Field Nat 114: 312 – 313.
